# Psychological wellbeing in Chinese university students: insights into the influences of academic self-concept, teacher support, and student engagement

**DOI:** 10.3389/fpsyg.2023.1336682

**Published:** 2024-01-16

**Authors:** Hua Zhang

**Affiliations:** College of Educational Science, Nanyang Normal University, Nanyang, China

**Keywords:** academic self-concept, teacher support, student engagement, psychological wellbeing, university students, mediation analysis justified

## Abstract

**Objective:**

This study investigates the complex interplay between academic self-concept, teacher support, student engagement, and psychological wellbeing among Chinese university students. We aimed to elucidate the mediating role of student engagement in these relationships.

**Methods:**

A sample of 597 Chinese undergraduate students from diverse universities participated in the study. We employed structured questionnaires to assess academic self-concept, teacher support, student engagement, and psychological wellbeing. Confirmatory factor analyses and structural equation modeling were used to test our hypothesized model.

**Results:**

Structural equation modeling indicated that the partial mediation model, which considered both direct and indirect effects, outperformed full mediation and direct effect models. Student engagement significantly mediated the relationships between academic self-concept, teacher support, and psychological wellbeing. Importantly, teacher support demonstrated a direct impact on psychological wellbeing, even when accounting for the mediating role of student engagement.

**Conclusion:**

This study underscores the pivotal role of student engagement as a mediator in the relationship between academic self-concept, teacher support, and psychological wellbeing among Chinese university students. While student engagement plays a substantial mediating role, our findings also recognize the persistent direct influence of teacher support on psychological wellbeing. These insights have implications for educators and policymakers aiming to enhance the wellbeing of university students by fostering positive academic self-concept and teacher support while recognizing the importance of student engagement.

## Introduction

As we have witnessed the rise of positive psychology in the last decade, researchers have primarily delved into individuals’ strengths and abilities that could result in ultimate functioning (Sheldon and King, 2001; Seligman et al., 2005). In fact, recently, positive psychology has flourished as one of critical fields encouraging and inspiring people to take action to improve performance and achieve objectives through placing strong emphasis on the positive aspects of their lives (Alex Linley et al., 2006; Wong, 2011; Lomas et al., 2021). In the field of positive psychology, it should be noted that researchers do not overlook the role of difficulties and setbacks, rather they significantly strengthen the existing attention to the positive aspects like enjoyment, self-efficacy, grit, satisfaction, engagement, enthusiasm, optimism, pleasure, interest, contentment, happiness, pride, and wellbeing (Fathi et al., 2023; Lim and Tierney, 2023).

Psychological wellbeing refers to a dynamic state by which one is able to enjoy his life and be satisfied with it, have a feeling of purpose and meaning, develop positive relationships with others, feel in control of his life, cope with the difficulties and stressor (Ryff and Singer, 2008: Luo and Hancock, 2020; Frost et al., 2022). Conceptualized as a wide-ranging concept, psychological wellbeing highlights the process by which one pursues his values and goals leading to personal growth and feelings of accomplishment (Ryff, 1989; Huppert, 2009; Trudel-Fitzgerald et al., 2019). As Diener and Ryan (2009) maintained, psychological wellbeing reflects the degree of contentment of students that is regulated based on the satisfaction and pleasure they drive from daily life, as well as their impression and attitudes regarding their wellness. It is postulated that the wellbeing of learners has a primary role that is greatly conducive to successful teaching and learning. Importantly, given its tight relationship with the quality of life and life satisfaction, students’ wellbeing in classrooms has gained increasing popularity among scholars and teachers (Mascia et al., 2020). Prior studies clearly show the positive correlation between students’ psychological wellbeing and their academic success and achievement (Kiuru et al., 2020; Cárdenas et al., 2022; Rehman et al., 2023). More specifically, in higher education, psychological wellbeing has been revealed to positively correlate with significant outcomes like better grades, positive behaviors, personal growth, and motivation (Capone et al., 2020). As Han (2021) indicated, university students’ wellbeing has significant advantages in the context of higher education in terms of enhancing learners’ ambitions, educational participation, academic achievement, and academic productivity. Regarding university students, psychological wellbeing can play a critical role in the learning process (Burris et al., 2009; Pan et al., 2023). Indeed, the wellbeing of students is linked to learners’ higher levels of educational achievement, determination, and enjoyment and lower levels of stress, as it can facilitate their confidence and resilience while learning (Morales-Rodríguez et al., 2020; Ngui and Lay, 2020; Yang, 2021). Taken together, therefore, it is critical to enhance our knowledge of the predictors of psychological wellbeing among learners, which have been seen as the essential source of learning achievement, particularly among the university students (Bewick et al., 2010; Li, 2022). More importantly, researchers have cautioned that poor wellbeing among students in higher education (i.e., university students) has become a profound issue that merits further investigation (Katajavuori et al., 2023). Appropriately, it is vital to address the antecedents of psychological wellbeing among students in higher education as an attempt to enhance this positive experience in classrooms.

In light of the growing number of studies exploring the psychological wellbeing among learners, there has been increased interest among scholars and researchers in identifying the predictors of this construct in general education contexts (Jeong et al., 2023; Slack and Priestley, 2023). Notwithstanding this, empirical investigations of the psychological wellbeing of university students are still rare and our knowledge regarding the precursors responsible for shaping it is missing from existing work. Indeed, greater care to underscore the influential determinant of wellbeing of students should be given by researchers, specifically in the context of higher education. Therefore, as an under-studied area of research, further studies are still required to broaden our understanding of students’ wellbeing, especially to identify the factors that contribute to this construct in higher education contexts. Accordingly, the current study represents an attempt to explore the predictive roles of academic self-concept, student engagement, and teacher support, as potential antecedents of university students’ psychological wellbeing. In addition, we examine for the very first time how academic self-concept, student engagement, and teacher support, as well as psychological wellbeing are associated together among university students.

## Review of the literature

### Psychological wellbeing

Emotions can play a rather essential role in the education domain (Cooke et al., 2006; Shao et al., 2013). Researchers in the field of positive psychology have developed crucial methods to yield insights into the paradigms of wellbeing (Vázquez et al., 2009; van Dierendonck and Lam, 2023). Psychological wellbeing can occur when people experience fewer negative emotions and more positive ones, and achieve more personal fulfillment (Siddiqui, 2015; Hatun and Kurtça, 2022). Psychological wellbeing can be characterized as the emotional state manifested through the prevalence of positive feelings and thoughts concerning the educational surroundings, educators, and fellow students (Fraine et al., 2005). It is suggested that psychological wellbeing is divided into two different aspects of intellectual (i.e., one’s own judgment about their personal fulfillment, in other words one’s satisfaction), and emotional (i.e., entailing one’s constructive and destructive emotions at the same time) (Salami, 2011; Morales-Rodríguez et al., 2020). As put forward by Kubzansky et al. (2023), psychological wellbeing pertains to one’s frequently seeking happiness, pleasure, and satisfaction in his everyday life, as well as experiencing positive emotions, while being resilient against negative emotions. Researchers often conceptualize psychological wellbeing in two different perspectives, namely the hedonic dimension which highlights the paramount role of pleasure and satisfaction, higher levels of positive affect, and lower levels of negative emotions, and eudaimonic dimension which underlines the significance of positive psychological functioning and personal development (Disabato et al., 2016; Joshanloo, 2016; Giangrasso, 2021; Greenier et al., 2021).

Extending this into education, student wellbeing has to do with the level of satisfaction and happiness derived from the educational environments by learners (Burris et al., 2009; Mclachlan and Justice, 2009; Hossain et al., 2023). According to Renshaw et al. (2015), student wellbeing is embedded in four main facets, including sense of connectedness, sense of efficacy, educational goal, and preference of studying. There is a general consensus among researchers that student wellbeing can be significantly conducive to learners’ academic outcomes in the classroom (Soutter et al., 2014; Siddiqui, 2015; Yang, 2021; Alqarni, 2022). Granted that prior studies have attached much importance to the critical role of student wellbeing in academic achievement, a number of sources of cognitive and psychological sources as antecedents of psychological wellbeing have been identified. For instance, in their study, Liu et al. (2021), factors such as ethnicity, stress, perceived worry, and social isolation were identified as negatively linked to psychological wellbeing, while emotional support, resilience, and physical health emerged as positive predictors among learners. Braun et al. (2020) explored the impact of teachers’ emotion regulation, burnout, and life satisfaction on students’ psychological wellbeing. Their results revealed that skills in emotion regulation, occupational health, and personal wellbeing significantly forecasted students’ wellbeing. Similarly, Rand et al. (2020) found that hope and optimism played a predictive role in the wellbeing of university students. All in all, although a bulk of research was previously done on students’ psychological wellbeing, relatively fewer studies have delved into the influence of psychological factors and personal traits of learners in shaping the wellbeing among university students, particularly in the higher education domain. Hence, as an attempt to bridge this research lacuna, the present study sought to investigate the predictive roles of academic self-concept, student engagement, and teacher support as the antecedents of university students in a university context.

### Academic self-concept

Shavelson et al. (1976) referred to self-concept as an individual’s perceptions regarding himself derived from his own experiences and judgments of an environment. Categorized into three structures of social, physical and academic, self-concept focuses on individuals’ perception of themselves (Wu et al., 2021). Social influences and self-attributions can be critical factors in explaining self-concept, and this in turn can act as an essential element in explaining and predicting one’s behavior in a specific context (Marsh and Martin, 2011; McInerney et al., 2012; Wimmer et al., 2019). Particularly, academic self-concept pertains to the extent a student feels he can learn effectively (Bong and Skaalvik, 2003; Kulakow, 2020; Arens et al., 2021). According to Arens et al. (2021), academic self-concept is conceived as learners’ feelings, attitudes, and reflections toward their own academic capacities. It is suggested that academic self-concept is often subject to the influence of a number of comparisons, namely social comparisons, dimensional comparisons, and temporal comparisons (Möller, 2005; Möller and Marsh, 2013). Social comparisons rest on the notion that learners compare their own academic success against that of peers in the same subject area. Dimensional comparisons are related to learners comparing their own academic success in a specific subject against their academic success in another subject area. Temporal comparisons focus on learners comparing their academic success in a specific subject area against their prior academic success in the same subject (Müller-Kalthoff et al., 2017; Wolff et al., 2018; Wolff and Möller, 2022).

Prior studies have ideated that academic self-concept is of paramount importance explaining and predicting behavior and wellness in classrooms and expectations about the professional future of students. For instance, in the context of Hong Kong, Wu (2012) examined the interplay between students’ self-concept and their psychological wellbeing in the higher education. Their findings demonstrated that higher levels of academic self-concept were associated with better psychological wellbeing among university students. Using a polynomial regression model, Schneider et al. (2022) tested the influence of academic self-concept on the wellbeing of students. Collecting data from a sample of 6,086 learners. The results provided empirical evidence to the claim that academic self-concept had a strong predictive role in shaping the psychological wellbeing among participants. in a similar attempt, Wikman et al. (2022) conducted a cross-sectional study to analyze the potential role of academic self-concept in predicting the wellbeing of students. To this aim, a total number of 143 took part in this study. The findings showed that self-concept positively correlated with the psychological wellbeing among learners.

Despite the recent growing attention, the exploration of the association between academic self-concept and psychological wellbeing among university students remains an area that requires further investigation. While some studies have touched upon the significance of academic self-concept in various educational settings, the specific connection between academic self-concept and psychological wellbeing in university students has not been extensively explored in prior research. Our study aimed to address this research gap by examining how academic self-concept might influence students’ psychological wellbeing within the context of higher education. Although there have been observations in diverse educational settings, a comprehensive exploration of this association within the specific context of university students’ wellbeing has been relatively limited.

### Teacher support

The intellectual and emotional bonds between teachers and students have long been conceptualized by researchers in a variety of frameworks (Jing-Qiu, 1997; Ansong et al., 2017). According to Knowles (1980), teachers play a multifaceted role in the learning process of students, assuming roles as procedural technicians, specialists, and connoisseurs. Teachers are encouraged to act more as catalysts and guides rather than solely as instructors or wizards. More importantly, teachers should act in a way to help students in their learning and acquisition, rather than making students learn (Liu et al., 2018; Lazarides et al., 2019; Greenier et al., 2023). In fact, teachers are of pivotal importance when it comes to creating, maintaining, and influencing a safe learning environment in which students can learn effectively (Walker and Kutsyuruba, 2019; Bishop et al., 2020; Valiente et al., 2020; Tvedt et al., 2021). As noted by Reeve and Shin (2020), teachers hold authoritative positions during teaching, enabling them to shape and guide the behaviors and attitudes of learners.

The concept of teacher support encompasses distinct dimensions that contribute to students’ educational experiences. Scholars have delineated two primary definitions elucidating teacher support within educational contexts. One facet, referred to as self-determination, hinges on the learner’s perception of support derived from experiences of cognitive, emotional, or autonomy-oriented assistance provided by the teacher in the classroom (Soenens and Vansteenkiste, 2005; Maulana et al., 2016; Banerjee and Halder, 2021; Chiu, 2021). This dimension emphasizes the student’s ability to recognize and interpret various forms of support received from the teacher, encompassing aspects of cognitive guidance, emotional nurturing, and encouragement toward self-directed learning. Conversely, the second dimension of teacher support encompasses a broader and more nuanced understanding. It encompasses two distinct aspects: a broad perspective highlighting continuous provision of informational, instrumental, emotional, or appraisal support to learners, and a narrower perspective focusing on the teacher’s ability to offer assistance, trust, foster friendships, and ensure satisfaction throughout the learning process (Lei et al., 2018; Romano et al., 2021). This broader definition encapsulates various forms of support, spanning from informational guidance to emotional reinforcement, thereby fostering a supportive environment conducive to holistic student development. In addition, Strati et al. (2017) proposed two forms of teacher support, namely instrumental and emotional support. The former pertains to teacher scaffolding (giving proper materials and feedback) that enables learners to effectively execute task and activities, while the latter focuses on teachers showing concern for their students’ affect and wellbeing and boosting their confidence in their capabilities to successfully complete the class activities.

Recent studies have shown evidence of the positive contribution that teacher support can offer to the learning process and wellbeing of students (e.g., Suldo et al., 2009; Brandseth et al., 2019; Guo et al., 2020; Hellfeldt et al., 2020; Zheng, 2022). Collecting data from a sample of 1228 Chinese students, Guo et al. (2020) investigated the association between teacher support ad students’ wellbeing. Their findings revealed that teacher support significantly correlated with the wellbeing of participants. In a cross-sectional research, Brandseth et al. (2019) investigated the relationship between teacher support and the wellbeing of learners. Administering a questionnaire to 574 students, the authors claimed that a supportive teacher can be a significant antecedent in shaping students’ wellbeing. Similarly, Suldo et al. (2009) carried out a study to identify the role of teacher support in students’ wellbeing. They provided evidence that student wellbeing was significantly subject to the effect of teacher support. In another study, Hellfeldt et al. (2020) also found a significantly positive association between teacher support and students’ wellbeing.

However, despite these studies, there remains a gap in the existing literature regarding a comprehensive exploration of the association between teacher support and student wellbeing. Although existing research has highlighted certain connections between teacher support and wellbeing, a thorough investigation integrating various dimensions of teacher support and their nuanced impact on the multifaceted aspects of student wellbeing within the higher education context appears to be underrepresented.

### Student engagement

Often conceptualized as multidimensional construct, student engagement has to do with students behavioral, cognitive, and emotional involvement within the learning process (Kahu and Nelson, 2018; Tight, 2020; Mohammad Hosseini et al., 2022). It is suggested that student engagement is embedded in four main dimensions, of behavioral engagement, cognitive engagement, emotional engagement, and social engagement (Appleton et al., 2006; Paloş et al., 2019; Bowden et al., 2021). Behavioral engagement is composed of a number of external observable behaviors including various facets of absenteeism, uncooperative and defiant behavior, withdrawal, following instructions, and learner involvement in classroom activities while adhering to the classroom norms and rules (Nguyen et al., 2018; Bouhafa et al., 2023; Rabi et al., 2023). Cognitive engagement relates to learners’ information processing, and is composed of deep and surface processing (Pintrich and Schragben, 2012; Li et al., 2023). While deep processing entails elaboration and organization, by which learners will be able to connect their prior knowledge with their current information, surface processing includes learners constantly reforming the information (Chen and Pedersen, 2012; Li and Lajoie, 2022; Derakhshan and Fathi, 2023). Emotional engagement is identified as the summative and enduring levels of emotions which learners experience, as well as the extent to which they have passion toward the activities (Bowden et al., 2021; Salta et al., 2022). Social engagement relates to students’ connection to and belongingness toward peers, staff, and teachers, which leads to a sense of community, belonging, purpose, socialization and interaction to others in the learning environment (i.e., bonding with classmates, arriving at school on time, and caring for and listening to others) (Wang and Hofkens, 2020; Yu et al., 2022).

Previous empirical studies have found that student engagement is related to positive personal outcome such as student wellbeing. For instance, by collecting data from 1174 students, Kareem et al. (2023) revealed that student engagement could positively exercise influence on the wellbeing of learners within the classroom. Enrolling a survey with 952 students, Bowden et al. (2021) delved int the relationship between student engagement and student wellbeing. Employing a structural model, their findings demonstrated that students’ engagement could significantly contribute to their psychological wellbeing. In a similar attempt, Yu et al. (2018) aimed to investigate the link between engagement and wellbeing among students. The findings indicated that student engagement positively correlated with learners’ wellbeing. Rodríguez-Fernández et al. (2018) also found that student engagement and their psychological wellbeing are positively and significantly related to each other. In another study, Kilgo et al. (2016) provided significant evidence that student engagement had an influential role in affecting the wellbeing among students. Pan et al. (2023) also unveiled that there was a positive association between student engagement and student psychological wellbeing.

While existing literature has extensively explored various facets of student engagement and psychological wellbeing, the specific relationship within the context of university students has remained relatively uncharted. However, there exists a gap in the exploration of the intricate interplay between engagement and the wellbeing of students within the university setting. Building upon prior research, this study endeavors to delve deeper into this underexplored territory. While acknowledging the valuable contributions of existing studies in related domains, it is pertinent to highlight that the specific nexus between university students’ engagement and their psychological wellbeing remains largely unexamined. The present study seeks to bridge this gap by pioneering an investigation into the potential role of university students’ engagement in shaping their psychological wellbeing.

### The current study

The primary aim of the current study is to investigate the direct and mediating relationships among academic self-concept, teacher support, student engagement, and psychological wellbeing among Chinese university students. The hypotheses put forth in this research are grounded in established theoretical frameworks and empirical evidence, seeking to contribute to the understanding of the intricate dynamics within the academic context.

**Hypothesis 1:** *Academic self-concept and psychological wellbeing.*

Our first hypothesis posits that academic self-concept is related to students’ psychological wellbeing. This proposition aligns with a substantial body of literature emphasizing the influential role of academic self-concept in shaping individuals’ perceptions of their abilities and competencies (Shavelson et al., 1976; Marsh and Martin, 2011). According to this theoretical framework, academic self-concept not only influences cognitive judgments but also plays a pivotal role in shaping emotional experiences within educational contexts (Salami, 2011; Kubzansky et al., 2023). Positive academic self-concept is associated with increased satisfaction, reduced negative emotions, and an overall sense of personal fulfillment (Wu et al., 2021; Hatun and Kurtça, 2022).

**Hypothesis 2:** *Teacher support and psychological wellbeing.*

The second hypothesis postulates that teacher support is related to students’ psychological wellbeing. This assertion is supported by various theoretical frameworks emphasizing the crucial role of teacher support in creating positive and supportive learning environments (Knowles, 1980; Reeve and Shin, 2020; Romano et al., 2021). Teachers, as influential figures in students’ academic lives, have the potential to provide emotional, instructional, and social support, fostering a sense of safety, trust, and belonging, ultimately positively impacting students’ emotional wellbeing (Suldo et al., 2009; Lei et al., 2018; Hernández-Sellés et al., 2019). In the context of higher education, where academic pressures are prevalent, teacher support becomes particularly significant (Han, 2021).

**Hypothesis 3:** *Mediating role of student engagement between academic self-concept and psychological wellbeing*.

The third hypothesis suggests that student engagement mediates the relationship between academic self-concept and psychological wellbeing. This hypothesis is grounded in the multidimensional construct of student engagement, which encompasses behavioral, cognitive, emotional, and social dimensions (Appleton et al., 2006; Kahu and Nelson, 2018). We posit that a positive academic self-concept enhances students’ motivation and willingness to engage in learning activities, leading to positive emotions, persistence, and a sense of purpose, ultimately contributing to enhanced psychological wellbeing (Wu, 2012; Arens et al., 2021).

**Hypothesis 4:** *Mediating role of student engagement between teacher support and psychological wellbeing.*

The fourth hypothesis proposes that student engagement mediates the relationship between teacher support and psychological wellbeing. This hypothesis is based on the premise that teacher support positively influences students’ emotional and cognitive engagement (Strati et al., 2017; Brandseth et al., 2019; Guo et al., 2020). Supportive teachers create an environment in which students feel cared for, motivated, and confident in their abilities, fostering active participation and emotional investment in learning experiences (Ansong et al., 2017; Reeve and Shin, 2020; Derakhshan et al., 2022). Consequently, we hypothesize that teacher support positively affects student engagement, which, in turn, contributes to higher levels of psychological wellbeing among university students.

In summary, these hypotheses are underpinned by established theoretical frameworks and empirical evidence, and their examination will provide valuable insights into the interplay between academic self-concept, teacher support, student engagement, and psychological wellbeing among Chinese university students.

## Materials and methods

### Participants

The participant pool for this study comprised 597 Chinese undergraduate students drawn from six diverse universities spanning across China. These participants, encompassing 219 (36.68%) males and 378 (63.31%) females, exhibited ages ranging from 19 to 26 years, with a mean age of 21.08 years (SD = 2.37). The inclusion of students from these six distinct universities aimed to capture a vivid perspective of the experiences and perceptions of Chinese undergraduate students on a broader scale.

Notably, these students represented a wide range of academic disciplines, showcasing the extensive diversity inherent in Chinese higher education institutions. Chinese universities offer a multifaceted array of undergraduate programs, encompassing fields such as engineering, humanities, natural sciences, social sciences, arts, business, and many more. These programs cater to a broad spectrum of interests and aptitudes, providing students with a wide-ranging choice to pursue their academic aspirations. The diverse academic landscape within Chinese higher education is characterized by a fusion of traditional disciplines and cutting-edge fields of study. Students have the opportunity to engage with innovative courses that blend contemporary knowledge with deep-rooted academic traditions. From disciplines fostering technological innovation to those preserving cultural heritage, Chinese undergraduate programs encapsulate a rich amalgamation of educational pursuits. This diverse academic milieu underscores the multifaceted nature of the educational experiences available to Chinese undergraduate students.

The selection of participants employed a convenience sampling technique, entailing the enlistment of individuals readily available and willing to engage in the research. This sampling approach was chosen for its practicality and efficiency, given the logistical constraints of conducting research with a large and geographically dispersed population of university students in China. Before gathering data, participants were briefed on the study’s objectives, the voluntary aspect of their involvement, and the confidential treatment of their responses. Each participant provided informed consent, and the study strictly adhered to ethical guidelines for research involving human subjects throughout its duration.

### Instruments

#### Psychological wellbeing

Psychological wellbeing was assessed using a shortened adaptation of the Psychological Wellbeing Scale originally developed by Ryff and Keyes (1995). This 18-item scale comprises six distinct facets, each capturing a specific dimension of wellbeing. The facet of autonomy evaluates self-determination and independence, encompassing an individual’s capacity to resist societal pressures and uphold personal beliefs. Environmental mastery gauges the ability to effectively navigate one’s surroundings and make choices that align with individual needs and values. Personal growth reflects the perception of ongoing personal development. Positive relations measure the presence of warm and trustworthy interpersonal relationships. Purpose in life examines one’s sense of purpose and direction in life. Finally, self-acceptance encompasses a positive self-attitude and favorable sentiments toward past experiences (Ryff and Keyes, 1995).

Each dimension was assessed using a set of three items, with participants expressing their degree of concurrence with associated statements on a 7-point scale, where 1 represented “strongly agree” and 7 denoted “strongly disagree.” For uniformity, responses to items were reversed, ensuring that elevated scores reflected enhanced psychological wellbeing. The 18-item scale’s robust reliability has been substantiated in prior research conducted with Chinese populations (Xu et al., 2020).

#### Academic self-concept

he evaluation of academic self-perception utilized the Academic Self-Description Questionnaire II (ASDQ II), an instrument crafted by Marsh (1990). Participants were tasked with gauging their academic competencies relative to their peers while providing responses to the questionnaire. Recorded on a five-point scale, responses spanned from 1 (strongly disagree) to 5 (strongly agree). Illustrative items encompassed statements such as “I demonstrate excellence in mathematics.” The questionnaire was specifically designed to probe students’ views on their accomplishments in distinct academic domains.

#### Teacher support

To gauge students’ perceptions of social support received from educators, the entire participant pool engaged with the 12-item teacher support scale formulated by Malecki et al. (2000). Embedded within this survey, three inquiries correspond to each distinct category of social support (Emotional, Instrumental, Appraisal, and Informational). Respondents encountered a prompt, starting with “My teacher(s)…,” followed by specific statements. For each statement, participants provided a dual response, indicating the frequency with which they encounter a particular supportive behavior and the importance of said behavior to them. Sample items include “My teacher(s) fosters an environment where questions are encouraged.” Participants utilized a 6-point Likert scale to denote their responses.

#### Student engagement

The evaluation of student engagement utilized Reeve’s (2013) validated Student Engagement Scale. Specifically designed for university-level assessment, this 17-item scale appraises four discrete facets of engagement: Agentic, Behavioral, Cognitive, and Emotional Engagement. Participants assigned ratings to each item on a 7-point Likert scale, ranging from 1 denoting “strongly disagree” to 7 signifying “strongly agree.” The selection of this tool was based on its capacity to holistically gauge diverse dimensions of student engagement within the higher education setting.

#### Procedure

In this study, a meticulously structured approach was undertaken to assemble a diverse cohort of Chinese undergraduate students representing various universities. The initiation of our process commenced with the diligent acquisition of ethical clearance from the pertinent institutional review board. This pivotal step underscored our unwavering commitment to upholding the rights and welfare of the participants involved in our study. Our recruitment methodology entailed a systematic engagement with academic departments and administrative entities across multiple esteemed universities dispersed throughout China. The primary objective was to solicit authorization for data collection activities within their respective academic precincts. Upon receiving the necessary endorsements from these esteemed institutions, a deliberate convenience sampling approach was employed. The utilization of convenience sampling facilitated the inclusion of participants who exhibited willingness and immediate availability to participate in our research endeavors. This approach was selected with careful consideration of logistical constraints and the optimization of available resources. It ensured an inclusive representation while adhering to the practicalities of participant involvement within the research framework.

Regarding participant compensation, it is important to note that participants in this study were not remunerated for their involvement. The data collection process was centered around voluntary participation, and participants did not receive any form of financial or material incentives for their contributions. The data collection itself revolved around a structured questionnaire administered to our participants. We set up data collection sessions in common university areas like libraries and student union buildings. This approach was chosen to ensure accessibility and convenience for our participants. During these sessions, trained research assistants were on hand to provide instructions and address any queries or concerns raised by the participants.

The data collection process, which included questionnaire administration, typically lasted between 25 to 35 min for each participant. This timeframe allowed participants ample opportunity to comprehensively complete the survey at their own pace. Throughout the research process, ethical considerations remained at the forefront of our efforts. We strictly adhered to principles such as informed consent, voluntary participation, and the safeguarding of confidentiality. Participants were made aware of their rights and the option to withdraw from the study at any point without facing any consequences. Our research team maintained the highest standards of data security and privacy to ensure that participants’ personal information remained confidential and secure. For data collection, we used a secure and user-friendly electronic platform. This platform facilitated the administration of the questionnaire digitally, enabling participants to respond using their own electronic devices, such as smartphones, tablets, or laptops. In cases where participants needed devices, we provided them. This electronic setup ensured data accuracy, efficient data management, and the automatic anonymization of responses.

#### Data analysis

In the initial phase of data analysis, we conducted a series of descriptive and correlation analyses to delve into the relationships among the variables of interest. These analytical procedures were carried out using the SPSS software, version 28.0.

To rigorously test our research hypothesis, we employed Structural Equation Modeling (SEM) within the Amos program, version 26.0. This approach allowed us to assess both the measurement model and the underlying structural model, in line with the methodology outlined by Blunch (2008). The assessment of our hypothesized model’s overall fitness was conducted using various fit indices, ensuring a comprehensive evaluation of model adequacy. These fit indices included the χ2-to-degree of freedom (df) ratio, the Goodness of Fit Index (GFI), the Comparative Fit Index (CFI), the Root-Mean-Square Error of Approximation (RMSEA), and the Standardized Root-Mean-Square Residual (SRMR).

To interpret these fit indices effectively, we adhered to established benchmarks. Specifically, a χ2/df ratio less than 3, accompanied by a *p*-value exceeding 0.05 (Chau, 1997), indicated a favorable goodness of fit. Furthermore, GFI and CFI values equal to or greater than 0.90 (Hu and Bentler, 1999) were considered indicative of a well-fitting model. Additionally, we deemed RMSEA values less than 0.08 and SRMR values below 0.10 as indicative of a good model fit, following the guidelines set by Vandenberg and Lance (2000).

## Results

Before proceeding with the main analyses, a thorough examination of the dataset was conducted to ensure the quality and integrity of the collected data (Hair et al., 2019). This initial data screening process involved assessments of normality, identification of missing values, and detection of potential outliers.

Normality of each variable was assessed using both visual and statistical methods. Histograms and Q-Q plots were inspected for visual indications of normal distribution. Additionally, the Shapiro-Wilk test was employed for a formal assessment of normality. Results indicated that the distribution of academic self-concept (*W* = 0.976, *p* = 0.120), teacher support (*W* = 0.963, *p* = 0.045), student engagement (*W* = 0.982, *p* = 0.274), and psychological wellbeing (*W* = 0.955, *p* = 0.032) did not significantly deviate from normality. The non-significant *p*-values suggest that assumptions of normality are tenable for subsequent analyses.

Missing values were examined for each variable to assess the completeness of the dataset following Little (1988). Results revealed a minimal amount of missing data, with less than 1% missing across all variables. Missing data were deemed to be missing completely at random (MCAR) based on Little’s MCAR test (χ^2^ = 6.45, df = 6, *p* = 0.38). To address missing data, a listwise deletion approach was employed, ensuring that only complete cases were included in the subsequent analyses.

Additionally, outliers were identified using a combination of visual inspection and statistical criteria. Boxplots were utilized to visually identify potential outliers for each variable. Additionally, the z-score method was applied, considering observations with z-scores beyond ± 3.29 as potential outliers. Outliers were detected in academic self-concept (*n* = 3), teacher support (*n* = 2), student engagement (*n* = 4), and psychological wellbeing (*n* = 1). Sensitivity analyses were conducted by comparing results with and without outliers, and no substantial changes in the overall pattern of results were observed. Consequently, outliers were retained in the dataset for further analysis.

Then the descriptive statistics and intercorrelations for the study variables were calculated. As seen in Table 1, the participants’ academic self-concept had a mean score of 3.92 (SD = 0.59) and exhibited high internal consistency (Cronbach’s α = 0.92). Teacher support was moderately rated, with a mean of 3.44 (SD = 0.63), and demonstrated good internal reliability (Cronbach’s α = 0.83). Student engagement had a mean score of 3.78 (SD = 0.91) and displayed excellent internal consistency (Cronbach’s α = 0.94). Psychological wellbeing was rated at a mean of 3.29 (SD = 0.76) and showed good internal reliability (Cronbach’s α = 0.87).

**TABLE 1:** Descriptive statistics.

	Mean	*SD*	Croanbach’s α	1	2	3	4
1. Self-concept	3.92	0.59	0.92	1			
2. Teacher support	3.44	0.63	0.83	0.34**	1		
3. Student engagement	3.78	0.91	0.94	0.45**	0.32**	1	
4. Wellbeing	3.29	0.76	0.87	0.39**	0.27**	0.49**	1

**p-value < 0.01.

Regarding the intercorrelations, academic self-concept was significantly positively correlated with teacher support (*r* = 0.34, *p* < 0.01), student engagement (*r* = 0.45, *p* < 0.01), and psychological wellbeing (*r* = 0.39, *p* < 0.01). Teacher support demonstrated significant positive associations with student engagement (*r* = 0.32, *p* < 0.01) and psychological wellbeing (*r* = 0.27, *p* < 0.01). Moreover, student engagement was positively correlated with psychological wellbeing (*r* = 0.49, *p* < 0.01).

Confirmatory factor analyses were executed to evaluate the singularity of latent factors, and three different measurement models were juxtaposed against the anticipated foundational model. Table 2 provides the fit indices for each model. The outcomes unveiled that, in comparison to alternative models, the envisioned four-factor measurement model exhibited more favorable alignment with the data (χ^2^ = 1134.114, df = 601, *p* < 0.001, CFI = 0.962, GFI = 0.891, RMSEA = 0.026, and SRMR = 0.039).

**TABLE 2:** The measurement models.

Measurement model	χ ^2^	df	CFI	GFI	RMSEA	SRMR
Single-factor model	1105.724	519	0.911	0.891	0.084	0.280
Two-factor model	997.283	513	0.944	0.936	0.071	0.235
Three-factor model	913.619	511	0.962	0.955	0.043	0.196
Four-factor model	842.307	508	0.971	0.968	0.036	0.165

Single-factor model: all the variables are treated as a unified factor. Two-factor model: wellbeing, student engagement, and self-concept are treated as a single factor, while teacher support is regarded as a distinct factor. Three-factor model: wellbeing and student engagement are amalgamated into one factor, while self-concept, and teacher support are treated as distinct factors. Four-factor model: Each of the variables is considered independently as separate factors.

Following the confirmation of the measurement model’s validity, the examination of alternative structural models ensued, scrutinizing our research hypotheses. More precisely, the envisaged partial mediation model underwent comparison with both a full mediation model and a direct effect model. The summary of fit indices for these three models is presented in Table 3. The direct effect model, which posited no mediating role for student engagement in the relationships between academic self-concept, teacher support, and psychological wellbeing, showed a significant chi-square statistic (χ^2^) of χ^2^(711) = 1360.423, *p* < 0.001. It demonstrated a commendable fit with CFI = 0.909, GFI = 0.836, RMSEA = 0.067, TLI = 0.891, and SRMR = 0.183.

**TABLE 3:** Results of structural models.

Model	χ ^2^	df	Δχ ^2^	GFI	CFI	RMSEA	TLI	SRMR
Direct effect model	1360.423**	711	–	0.836	0.909	0.067	0.891	0.183
Full mediation model	1156.312**	707	204.111	0.889	0.960	0.042	0.937	0.074
Partial mediation model	898.211**	702	258.101	0.912	0.976	0.036	0.967	0.061

Δχ^2^ indicates the differences between models.

**p-value < 0.001.

In contrast, the full mediation model, proposing complete mediation through student engagement, exhibited χ^2^(707) = 1156.312, *p* < 0.001, with superior fit indices: CFI = 0.960, GFI = 0.889, RMSEA = 0.042, TLI = 0.937, and SRMR = 0.074. The hypothesized partial mediation model, accounting for both direct and indirect effects, yielded χ^2^ (702) = 898.211, *p* < 0.001, with excellent fit indices: CFI = 0.976, GFI = 0.912, RMSEA = 0.036, TLI = 0.967, and SRMR = 0.061.

Comparatively, both the full mediation model and the partial mediation model outperformed the direct effect model in terms of goodness of fit. These findings substantiate our hypothesis that student engagement plays a substantial mediating role in the relationships involving academic self-concept, teacher support, and psychological wellbeing among university students.

**FIGURE 1:**
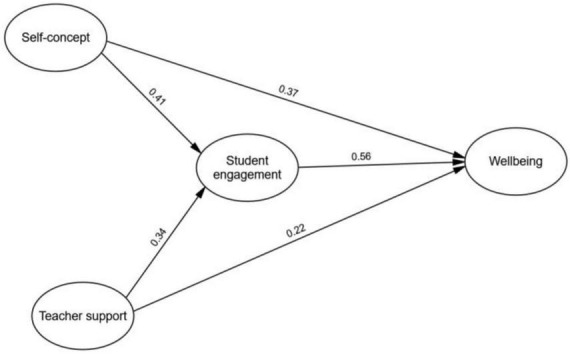
The mediation model.

Figure 1 presents the path and parameter estimates for the ultimate fit model, which corresponds to the partial mediation model. As illustrated in Figure 1, all path coefficients within the model reached statistical significance. Notably, the structural model revealed significant direct relationships among the key variables. Specifically, academic self-concept exhibited a substantial and direct positive association with student engagement (β = 0.41, *p* < 0.001), underscoring its significant impact on students’ engagement levels. Similarly, teacher support displayed a significant and direct positive relationship with student engagement (β = 0.34, *p* < 0.01), highlighting the role of teacher support in fostering students’ engagement. Furthermore, it is noteworthy that student engagement demonstrated a robust positive link with student wellbeing (β = 0.56, *p* < 0.001), emphasizing its pivotal role as a positive predictor of students’ overall wellbeing.

In our study, we utilized Baron and Kenny (1986) mediation analysis approach to explore whether student engagement played a mediating role in the relationships among the examined variables. The outcomes, as depicted in Table 4, offer insights into this mediation process.

**TABLE 4:** Path estimates of structural model.

Path estimates (*t*-value)			
	Direct model	Full mediation model	Partial mediation model
Self-concept → Wellbeing	0.39 (6.92***)		0.37 (6.88***)
Teacher support → Wellbeing	0.19 (4.43**)		0.22 (4.71**)
Self-concept → Engagement		0.44 (7.82***)	0.41 (7.69***)
Teacher support → Engagement		0.32 (6.09***)	0.34 (6.37***)
Engagement → Wellbeing		0.59 (9.41***)	0.56 (9.21***)

Engagement: student engagement.

**p < 0.01,

***p < 0.001.

In the direct effects model, we observed noteworthy path coefficients between academic self-concept and wellbeing (β = 0.39, *p* < 0.001) and between teacher support and wellbeing (β = 0.19, *p* < 0.01). These findings correspond to the initial step in Baron and Kenny’s mediation analysis, confirming significant direct links between the independent variables and the dependent variable.

Subsequently, in the full mediation model, we identified substantial path coefficients between academic self-concept and student engagement (β = 0.44, *p* < 0.001) and between teacher support and student engagement (β = 0.32, *p* < 0.001), which substantiates the second step in the mediation process. Furthermore, it is worth noting that student engagement exhibited a robust positive association with wellbeing (β = 0.59, *p* < 0.001), indicating its vital role as a mediating factor in the relationship between academic self-concept, teacher support, and psychological wellbeing.

Within the partial mediation model, self-concept is directly related with wellbeing β = 0.37 (*t* = 6.88, *p* < 0.001). Likewise, the path coefficient from teacher support to psychological wellbeing is β = 0.22 (*t* = 4.71, *p* < 0.01), denoting a statistically significant direct relationship. This finding suggests that teacher support has a direct impact on wellbeing, even when considering the influence of student engagement. Moreover, in the direct effects model, the path coefficient for teacher support to psychological wellbeing is β = 0.19 (*t* = 4.43, *p* < 0.01), indicating a significant direct relationship. This serves as a reference point for understanding the partial mediation process.

The presence of significant direct paths from teacher support to wellbeing in both the partial model and the direct model signifies partial mediation. In essence, while student engagement acts as a mediator, it does not entirely account for the relationship between teacher support and psychological wellbeing. This underscores the complex nature of these variables, with student engagement playing a substantial mediating role, while acknowledging the ongoing direct influence of teacher support on wellbeing. This observation highlights the interrelationships among these factors, emphasizing the crucial role of student engagement as a mediator, while also recognizing the persistent direct impact of teacher support on wellbeing.

Finally, to mitigate potential common method bias, we executed Harman’s single-factor test, encompassing all latent variables evaluated through self-reported measures—namely, academic self-concept, teacher support, student engagement, and psychological wellbeing. Analysis outcomes disclosed that the initial factor elucidated 36.08% of the variance, falling beneath the 50% threshold and affirming the absence of common method bias in our investigation.

## Discussion

The current study sought to test the predictive role of academic self-concept, student engagement, and teacher support in influencing the psychological wellbeing among university learners. A number of significant findings were put forward through the current research. Regarding the proposed structural model and the formulated hypotheses for the purposes of the current research, the main results are able to offer important implications for the association between/among these variables.

Firstly, the results demonstrated that academic self-concept positively predicted the psychological wellbeing among learners, that is students holding a positive academic self-concept, characterized by a belief in their academic capacities and capabilities, showed higher levels of psychological wellbeing. The significant effect of academic self-concept on students’ wellbeing is in accordance with a number of past research undertakings in various educational settings (e.g., Wu, 2012; Schneider et al., 2022), which revealed that students’ self-concept in an academic environment is significantly conducive to their psychological wellbeing. Furthermore, the findings are parallel to the results of the Wikman et al. (2022) study indicating that there was a positive and strong association between academic self-concept and engagement among students. This finding might be justified in light of the fact that those learners endorsing stronger self-concepts about their own academic competencies are more likely to be resilient in the face of setbacks and challenges in the classroom (McInerney et al., 2012; Haktanir et al., 2021; Huang et al., 2021), and this in turn might lead to experiencing higher levels of wellbeing while learning. This indicates that when students perceive themselves as adept and proficient in their academic pursuits, they are inclined to encounter elevated life satisfaction, happiness, and a sense of purpose—integral components of psychological wellbeing. Furthermore, the association between academic self-concept and wellbeing finds support in self-determination theory (Deci and Ryan, 1985). As per this theoretical framework, individuals harboring a positive self-concept in a particular domain, such as academics, are predisposed to experience intrinsic motivation and heightened psychological wellbeing (Deci and Ryan, 2000). When students have confidence in their academic capabilities, they are more likely to actively participate in learning activities voluntarily, which can lead to a sense of accomplishment and overall wellbeing (Marsh and Martin, 2011). Additionally, the literature on academic self-concept has consistently shown its association with other positive academic outcomes, such as higher academic achievement and motivation (Bong and Skaalvik, 2003). These positive academic outcomes can, in turn, contribute to students’ overall sense of wellbeing.

Secondly, the results demonstrated that student engagement positively exerted impact on university students’ wellbeing. In other words, higher levels of student engagement, denoting active involvement and enthusiasm in learning activities, were associated with better psychological wellbeing in university students. The results of our study add support to the increasing body of literature demonstrating that student engagement has significant advantages for learners including greater levels of psychological wellbeing (e.g., Kilgo et al., 2016; Paloş et al., 2019; Bowden et al., 2021; Kareem et al., 2023). It can be argued that engaged students are inclined to be more confident, empowered, and committed educationally and cognitively, which could help them in the process of personal growth and development, as well as in forming positive and proactive habits of mind, thereby they are more likely to feel psychological wellbeing (Boulton et al., 2019). This finding is also on a par with previous literature showing that there is a positive association between student engagement and student psychological wellbeing (e.g., Siddiqui, 2015; Rodríguez-Fernández et al., 2018; Yu et al., 2018; Pan et al., 2023).

Thirdly, our findings underscore the significant role of teacher support in influencing the psychological wellbeing of university students, indicating that heightened perceptions of support from educators contribute to enhanced psychological wellbeing. These outcomes align with prior research, exemplified in studies such as those conducted by Suldo et al. (2009) and Brandseth et al. (2019), which establish teacher support as a predictive factor influencing students’ wellbeing. Consistent evidence suggests that students who perceive their teachers as supportive, caring, and empathetic are more prone to experiencing heightened emotional wellbeing (Suldo et al., 2009; Soutter et al., 2014; Guo et al., 2020; Zheng, 2022). This discovery underscores the pivotal role of the teacher-student relationship as a substantial determinant in shaping the overall mental health and wellbeing of students (Holfve-Sabel, 2014). Also, our findings support the empirical research of Guo et al. (2020) and Hellfeldt et al. (2020) related to the relationships between teacher support and students’ psychological wellbeing. A possible explanation seems valid in this regard. When teachers are able to regulate their negative emotions in classroom and provide the necessary encouragement and feedback while teaching, they can create a pleasant learning environment in which students’ sense of happiness is enhanced and boosted. This in turn, may result in higher levels of psychological wellbeing among students (Zheng, 2022).

Moreover, it is well-established that teacher support can enhance students’ motivation and engagement in learning (Reeve and Shin, 2020). When students feel that their teachers are genuinely interested in their success and wellbeing, they are more likely to be motivated to participate actively in class and take ownership of their learning process (Holfve-Sabel, 2014). This heightened engagement can lead to a sense of accomplishment and satisfaction, contributing to overall psychological wellbeing (Burris et al., 2009; Yu et al., 2018). The constructive correlation between teacher support and student wellbeing might find elucidation through self-determination theory (Deci and Ryan, 2000). This theoretical framework posits that when students discern autonomy, competence, and relatedness in their learning milieu—all nurtured by teacher support—they are predisposed to encounter intrinsic motivation and psychological wellbeing. In this context, teacher support nurtures students’ sense of relatedness, a pivotal psychological need intricately tied to overall wellbeing.

In addition, it was revealed that student engagement mediated the relationships between academic self-concept and psychological wellbeing of Chinese university students. This finding aligns with a substantial body of literature emphasizing the central role of student engagement in academic success and wellbeing (Yu et al., 2018; Boulton et al., 2019; Kareem et al., 2023). When students are actively involved in their learning, they are more likely to experience a sense of purpose, fulfillment, and accomplishment. This positive emotional experience is a key component of psychological wellbeing (Cooke et al., 2006; Rodríguez-Fernández et al., 2018). It can be argued that when students possess a positive academic self-concept, encompassing beliefs about their competence and proficiency in the academic realm, there is a heightened likelihood of experiencing intrinsic motivation (McInerney et al., 2012). This intrinsic motivation, in a cascading effect, results in increased involvement in educational pursuits. As students become more profoundly engaged in their academic endeavors, they are prone to encounter an enhanced sense of achievement and contentment, thereby contributing positively to their overall wellbeing (Morales-Rodríguez et al., 2020).

Moreover, SEM results confirmed the mediating role of student engagement in the relationships between teacher support and psychological wellbeing. This finding resonates with the extensive body of literature emphasizing the pivotal role of teacher support in students’ wellbeing (Suldo et al., 2009; Holfve-Sabel, 2014; Guo et al., 2020). It underscores the idea that when teachers provide support—both academically and emotionally—it can have far-reaching effects on students’ psychological wellbeing. Teacher support can create a conducive learning environment where students feel valued, secure, and motivated, ultimately leading to enhanced wellbeing. Moreover, the mediating role of student engagement highlights the importance of teacher-student relationships and support in educational settings. It underscores that teacher support is not merely about academic assistance but also about creating an environment where students are emotionally invested and actively participate in their learning (Brandseth et al., 2019). Teachers who establish strong connections with their students and provide guidance, encouragement, and empathy can facilitate a deeper level of engagement, which, as this study shows, is closely linked to wellbeing (Zheng, 2022).

Overall, our study contributes significantly to the existing literature by highlighting the predictive role of academic self-concept, student engagement, and teacher support in influencing the psychological wellbeing of Chinese university students. Notably, our findings affirm the positive associations between academic self-concept, student engagement, and teacher support with psychological wellbeing, thereby providing empirical support in a Chinese university context. Additionally, this study extends prior research by delineating the mediating roles of student engagement in the relationships between academic self-concept, teacher support, and psychological wellbeing. By validating these relationships within the specified academic setting, our study emphasizes the interplay among these constructs, elucidating how they collectively contribute to fostering students’ psychological wellbeing in higher education contexts. These novel insights underscore the multifaceted nature of student wellbeing, emphasizing the significance of academic self-concept, student engagement, and teacher support as integral facets in promoting and enhancing the psychological wellbeing of university learners.

## Conclusion

In summary, this study has contributed notable insights into the extant associations between academic self-concept, teacher support, student engagement, and psychological wellbeing among Chinese university students. Our findings support the hypothesis that student engagement significantly mediates the relationships between academic self-concept, teacher support, and psychological wellbeing. Additionally, teacher support has been shown to have a direct impact on psychological wellbeing, even when considering the mediating role of student engagement. The results emphasize the crucial role of student engagement as a mediator in this multifaceted relationship. Student engagement not only directly influences psychological wellbeing but also acts as a bridge that connects academic self-concept and teacher support with wellbeing. This underscores the dynamic nature of the student experience and highlights the importance of fostering active engagement among university students.

These findings provide valuable insights for educators, policymakers, and practitioners committed to enhancing the wellbeing and academic achievements of university students. First and foremost, educators hold a pivotal role in nurturing students’ academic self-concept, a foundational element for their engagement and overall wellbeing. Encouraging a growth mindset and providing opportunities for skill development and mastery can significantly contribute to fostering a positive academic self-concept. Moreover, the direct impact of teacher support on psychological wellbeing emphasizes the critical need for creating supportive learning environments. Both teachers and educational institutions should prioritize the provision of emotional, instructional, and social support to students. Additionally, offering professional development programs for educators can equip them with effective strategies for providing this support. Recognizing the mediating function of student engagement, educators and institutions should actively cultivate engagement strategies within classrooms. This encompasses the creation of interactive learning experiences, the encouragement of active participation, and the establishment of a sense of community within educational settings.

Viewing student wellbeing as an essential marker of educational quality, policymakers within higher education are urged to prioritize its recognition. It is vital to move beyond mere acknowledgment, actively advocating and supporting the augmentation of both academic self-concept and teacher support, integral components that significantly bolster student engagement and foster psychological wellbeing. To facilitate these advancements, policymakers ought to adopt multifaceted interventions geared toward cultivating a supportive academic milieu. Comprehensive teacher training programs serve as a cornerstone, equipping educators with the requisite emotional, instructional, and social support skills. By fostering positive teacher-student relationships and cultivating inclusive learning environments, these programs play a pivotal role in nurturing supportive teaching practices. Complementing such programs, establishing robust institutional frameworks that prioritize student mental health and wellbeing becomes paramount. Initiatives such as counseling services, mentorship programs, and peer support networks woven into the fabric of universities provide critical avenues for students to seek guidance, support, and encouragement, further solidifying their wellbeing within the educational realm.

A strategic integration of wellbeing and self-development modules into the curriculum represents another avenue for transformative change. These modules, designed to promote self-reflection, resilience, and personal growth, empower students to develop a positive academic self-concept. Equipped with stress management techniques, bolstered self-efficacy, and the tools for maintaining a healthy work-life balance, students become better poised to navigate the challenges of academia. Parallel to curriculum adjustments, the implementation of regular assessments aligned with feedback mechanisms is pivotal. These assessments gauge student satisfaction, wellbeing, and engagement levels, providing students with avenues to voice concerns and actively participate in shaping the learning environment. Such mechanisms reinforce the commitment to student-centered educational approaches, facilitating continuous improvements reflective of students’ evolving needs.

Simultaneously, investing in research initiatives aimed at understanding the nuanced challenges faced by students in higher education is imperative. These insights drive the allocation of resources toward tailor-made solutions, catering to the diverse needs of students across various academic disciplines. Research-driven interventions are pivotal in effecting nuanced changes that resonate deeply within the student body. Embedding these multifaceted strategies within higher education policies engenders an environment valuing not only academic attainment but also prioritizing the holistic wellbeing of students. Collaborative efforts among policymakers, academic institutions, mental health professionals, and student representatives are integral for the effective implementation and evaluation of these policies. Through concerted action, these strategies have the potential to sculpt a thriving ecosystem that nurtures resilient, engaged, and psychologically healthy individuals, enriching the educational landscape for generations to come.

Looking ahead, this study lays the groundwork for further exploration of the intricate relationships among these variables. Subsequent research endeavors could delve into the influence of additional factors, such as cultural variations, on shaping these relationships. Moreover, longitudinal studies may offer valuable insights into the development of student engagement and its enduring effects on wellbeing. This ongoing research can contribute to a deeper understanding of how to optimize the learning environment for the benefit of students’ holistic development and success.

Nevertheless, it is imperative to acknowledge several limitations inherent in this study that warrant consideration when interpreting the results. Firstly, the adoption of a cross-sectional design, capturing data at a singular time point, hinders the establishment of causal connections among variables. For a more nuanced comprehension of the intricate dynamics and causal orientations of these relationships across time, a longitudinal research approach would be indispensable. Another limitation pertains to potential sampling bias, as the study exclusively targets Chinese university students. As a result, the applicability of the findings to other student cohorts, both within and beyond China, may be circumscribed. Cultural, regional, and demographic distinctions could introduce variability into the observed relationships, necessitating recognition of this potential bias.

Moreover, the data gathering process hinges on self-report surveys, introducing susceptibility to response bias and social desirability bias. Respondents might furnish responses they deem socially acceptable or congruent with their self-concept, introducing a potential influence on result accuracy. Also, another inherent limitation of this study involves potential variability in responses due to the use of diverse electronic devices by participants, which was not explicitly examined for its impact on survey responses. Future research endeavors could address this limitation by conducting a thorough investigation into the potential impact of diverse electronic devices on survey responses, implementing controls or standardized procedures for device usage, and validating data consistency across various electronic mediums to ensure robustness and reliability in findings.

Measurement validity is another concern, as the study employed structured questionnaires to assess academic self-concept, teacher support, student engagement, and psychological wellbeing. It is crucial to ensure that these measures are psychometrically sound and culturally appropriate for the target population to maintain the validity of the results. Furthermore, while the study identifies student engagement as a mediator between academic self-concept, teacher support, and psychological wellbeing, this mediation model simplifies the complex interplay of factors contributing to psychological wellbeing. Other variables not considered in this study may also play significant roles. The study does not delve into specific contextual factors that may influence the examined relationships, such as academic curriculum, institutional policies, and extracurricular activities. These factors can impact students’ self-concept, teacher support, engagement, and wellbeing, and future research could benefit from exploring these contextual elements. Also, it is acknowledged that other aspects such as peer relations, family dynamics, and cultural factors or additional socio-environmental factors could also significantly contribute to the broader construct of student wellbeing but were not explicitly examined within the scope of this research. Future studies should endeavor to encompass a broader spectrum of factors to provide a more comprehensive understanding of the diverse facets influencing student wellbeing within educational contexts.

Moreover, the study does not explore potential reverse or bidirectional relationships. While it acknowledges the direct impact of teacher support on psychological wellbeing, it does not investigate how students’ wellbeing or engagement levels might influence teacher support. The abstract also lacks information about the response rate and details regarding the representativeness of the sample. A low response rate or specific participant characteristics could affect the external validity of the findings. Finally, the study does not address the potential temporal stability of the variables. Psychological wellbeing, self-concept, and engagement can vary throughout a student’s academic journey, and the study does not account for this variability.

## Data availability statement

The raw data supporting the conclusions of this article will be made available by the authors, without undue reservation.

## Ethics statement

The studies involving humans were approved by the College of Educational Science, Nanyang Normal University, Nanyang City, China. The studies were conducted in accordance with the local legislation and institutional requirements. The participants provided their written informed consent to participate in this study.

## Author contributions

HZ: Conceptualization, Data curation, Formal Analysis, Funding acquisition, Investigation, Methodology, Project administration, Resources, Software, Supervision, Validation, Visualization, Writing – original draft, Writing – review and editing.
